# On the methodology of conventional and semi-system formwork project comparison

**DOI:** 10.1016/j.mex.2024.102824

**Published:** 2024-06-29

**Authors:** Muhammad D.V. Lawdy, Eva Arifi, Ming Narto Wijaya, Bagus Krisnawan, Taufiq Rochman

**Affiliations:** a Civil Engineering Department, Gedung A Teknik Sipil, Brawijaya University, Jl. MT. Haryono 169, Kec. Sukun, Malang 65146 Indonesia; b Civil Engineering Department, Gedung Teknik Sipil, State Polytechnic of Malang, Jl. Soekarno Hatta no. 9, Kec. Lowokwaru, Malang 65141 Indonesia

**Keywords:** Formwork, Value engineering, Beam, Column, And slabs, *Comparison of Conventional and Semi-System Formwork*

## Abstract

Formwork refers to a temporary structure or mold that is used to hold concrete in place from the moment it is poured until it reaches the desired level of strength. Efficient utilization of formwork is a crucial aspect to take into account. This study aims to analyze the costs related to column, beam, and floor slab formwork by employing value engineering. The analysis will specifically compare the use of conventional formwork with semi-system formwork. The findings of this study propose the substitution of the column and beam formwork due to the substantial expenses associated with the conventional formwork utilized in this structure. By utilizing semi-system formwork, the expenses associated with beam formwork can be diminished by 47 %. It is advisable to employ semi-system formwork for column formwork as it can result in a cost reduction of 37 %. However, it is recommended to continue using traditional formwork for floor slab construction, as it is still 15 % more cost-effective per square meter compared to using semi-system formwork.

• This method highlights the limitations of traditional formwork.
• This method investigates the implementation of semi-system formwork in construction projects.
• This study aims to examine and compare conventional and semi-system formwork projects.

## Graphical abstract

**Figure:**
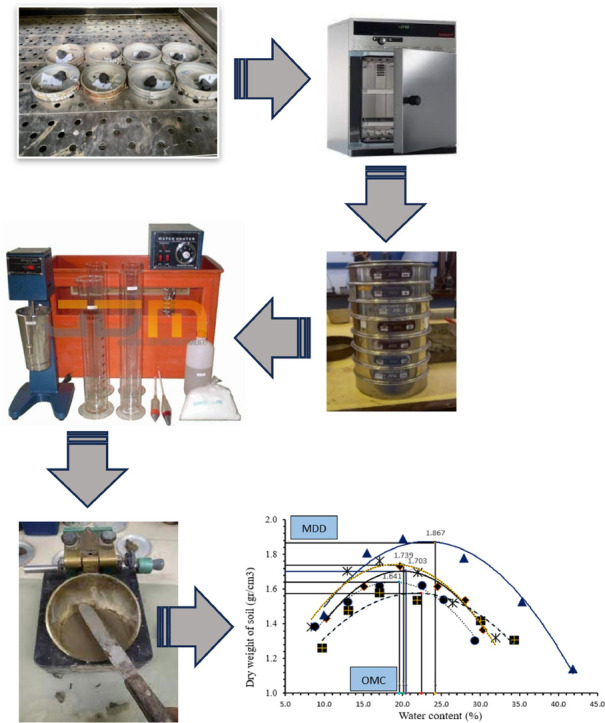

Specifications table

**Table:** 

Subject area:	*Civil Engineering*
More specific subject area:	*Value Engineering of Management Construction*
Name of your method:	*Comparison of Conventional and Semi-System Formwork*
Name and reference of original method:	*Das, R., Bhattacharya, I., & Saha, R. (2016). Comparative Study between Different Types of Formworks. International Research Journal of Advanced Engineering and Science (21).*http://irjaes.com/wp-content/uploads/2020/10/IRJAES-V1N4P210Y16.pdf
Resource availability:	*N/A*

## Background

The construction methods or systems utilized have experienced annual growth in response to the expansion of the Indonesian building industry. Generally, individuals necessitate buildings that are both secure and cost-effective, as well as efficient and straightforward to erect. During a period of intense global competition, multiple industries are affected [1]. Numerous enterprises struggle to maintain their operations when faced with competition. Competition has emerged in various sectors, including the construction industry. It is utilized in various construction projects of varying scales, ranging from minuscule to colossal. These projects are being carried out in response to the increasing demands of businesses and communities for large-scale engineering construction, housing, and structures [2,3]. In light of the growing demand, it is imperative for the construction industry to enhance its efficiency in order to minimize construction time and costs while upholding quality standards [4].

Planning and scheduling are crucial elements of project management as they govern the efficiency and performance of a project. These two components provide a fundamental basis or principle that can be utilized or executed, representing two separate stages in the process of estimating the duration of a project [5,6]. Essentially, it is not possible to separate existing obstacles from the potential to create delays in a project that is already in progress. Not completing a project within the designated timeframe can result in wasted time when implementing its outcomes, leading to delays or postponements in the application of the project's development results [7,8]. Conversely, a project's long-term viability will be enhanced by thorough planning, while it will be negatively impacted by insufficient planning [9]. Effective planning methodologies are essential for accurate execution and careful evaluation of site-specific factors in the field of building construction. A correlation exists between quality, cost, time, and specification in the field of construction management [10]. Three essential components must be designed in building projects as they significantly impact the success of structural work. The three components consist of a composite concrete, concrete bars, and cisterns, with the latter being particularly costly [11]. Globalization has accelerated the velocity and accuracy of information dissemination across international boundaries and facilitated the widespread adoption of innovative and creative technology among the global population. This has led to advancements in global construction practices in multiple ways. Concrete is molded into the desired shape and maintained under pressure by a temporary structure called a cistern. It provides support for wet cement during the process of hardening, which is a crucial step in the formation of concrete. Braces constitute approximately 20–25 % of the overall construction expenses, making them a crucial element. Contemporary case techniques enable the faster production of larger formwork components, leading to reduced labor and time requirements overall [12].

## Method details

Construction projects often incur substantial implementation costs on-site due to the expenses linked to inadequate building planning and design. Planning involves the selection of materials that will ultimately determine the approach and quality of execution. Therefore, it is necessary to optimize costs. Qualifying can be completed either during the planning phase or while conducting actual fieldwork. Employing feedback analysis based on building planning and design data can optimize construction costs. The objective of this research is to present alternative options that can be compared to the original designs with the aim of optimizing building expenses. Value engineering analysis is the strategy employed to reduce construction costs.

Construction projects employ systematic approaches during the design and construction phases. Multidisciplinary teams are used to generate innovations that enhance quality, performance, functionality, and cost effectiveness throughout the project's lifetime. An obstacle to the implementation of value engineering in Indonesia is the lack of knowledge among various stakeholders about the technique [13].

Each task has the capacity to enhance output and productivity, and the worth of the savings derived from value engineering is assessed in relation to the estimated initial cost. Value Engineering Implementation (VE) is a method that optimizes project cost efficiency by maximizing the value for money when designing building structures. If there are indications of inefficiency in the utilization of the project budget, it is advisable to follow this recommended approach for its completion [14].

This study will analyze the influence of value engineering on the structural aspects of the building construction project. The project was chosen as a study subject due to its potential to achieve a more cost-effective design, reduce unnecessary expenses, and deliver greater overall value, as perceived by the building's developer or owner. Costly consequences will arise from poor decisions made during the construction process, regardless of the factors of use, age, quality, or appearance. The analytical method of value engineering was selected for the structural construction due to the significant cost associated with building structures, amounting to IDR 23,093.24 million. The cost will be decreased by evaluating each option and examining Pareto diagrams using the Value Engineer stages - information, speculation, and presentation. The cost of the building work, which is comparatively high compared to other work prices, will be reduced by eliminating wasteful expenses [15]. This study utilizes value engineering to optimize the cost and functionality of the construction of the building object, encompassing both practical components and aesthetic functions. This approach is necessary as numerous materials do not serve the interests of the owners or users. This study aims to quantify the magnitude of cost reductions achieved through the implementation of the value engineering technique.

The choice of materials for embroidery varies depending on the type of construction, whether it is commercial, residential, industrial, or any other category. Choosing the cistern is a crucial task that directly affects the total cost of the project. It is a crucial element that also impacts the design of buildings. This study will investigate the selection of cistern materials for various architectural typologies [16]. A formwork is a useful tool for molding concrete into the desired sizes, shapes, and positions. During the investigation, both conventional and semi-system beech containers were evaluated [17]. A fork can be utilized to precisely mold, dimension, or position concrete. A comparative analysis was conducted to assess the differences between the system method and the semi-system cushioning approach for beams and floor slabs [18]. Constructing floor slabs is a labor-intensive task. This is because there are significantly more floor slabs involved in this task compared to any other task. The floor slabs of a building are a costly yet optimizable component of construction. Construction projects frequently employ conventional techniques, which involve the utilization of timber beams, cisterns, and supports. This particularly applies to projects that involve concrete structures. With rapid technological advancements, novel concepts are arising to enhance both the quantity and caliber of work [19]. Bags made of steel, aluminium, wood, or prefabricated shapes are commonly employed for the purpose of pouring concrete. The formwork used for pouring larger building elements such as columns, beams, slabs, and sliding walls is also used for smaller building components like stairs. When selecting cisterns for advanced development, it is crucial to consider the factors of cost, time, and quality. The conventional formwork method is a frequently employed technique for constructing concrete buildings. In the traditional method of constructing a cistern, standard hardwood panels relate to wooden frames on the rear to provide support for the weight and horizontal structure of wet concrete [20]. As a result, the traditional casing has been substituted in the casing system with permanent casing, which can be taken out after use and incorporated into structural elements to provide overall structural strength to the system. On the other hand, due to the immense magnitude [21].

This study aims to assess the cost per square meter of construction by analyzing and contrasting various methods for structural work, drawing on previous research. The objective of this analysis was to identify the optimal approach for setting up the cistern and to negotiate a lower initial cost for the required construction work. This investigation primarily centers around the construction of the Psychology Building. This study examined various options for crates to identify alternatives to structural work that are more readily accessible, cost-effective, versatile, and efficient. This would facilitate a significant enhancement in productivity without compromising the quality of the concrete. The objective of the study is to assess the cost per square meter of each installation.

## Comparative of formwork methods

This study employs a qualitative research methodology conducted within the Psychology lecture hall. The term used to describe a post-positivist observational approach is qualitative method [22]. The objective of qualitative observation is to recognize and address intangible attributes, such as emotions, thoughts, experiences, and others [23]. This method utilizes the observer as the primary instrument for assessing the state of natural entities. Qualitative research is characterized using triangulated (mixed) data collection methods, inductive data inspection, deliberate sample data collection, and a focus on meaning rather than generalization.

This comprised data on the predominant formwork types employed in construction, the circumstances in which original functions can be substituted with alternative materials without compromising them, and the frequency of repeated utilization of each replacement cushion. The study gathered secondary data, such as Surabaya pricing lists for commodities and labor, budget plans, technical specifications, implementation methods, and work images.

Subsequently, the data is aggregated to ascertain which task results in the highest expenditure. Next, the Pareto diagram should be examined to assess the suitability of a value engineering application, and subsequently, the cost model should be resolved. The findings of the Pareto diagram suggest that work which incurs a substantial cost is considered valuable work that surpasses 80 % of the total effort. In such cases, it is necessary to engage value engineers to carry out the tasks. Specifically, the original material is substituted with more affordable alternatives while maintaining the same level of strength and quality in the structure. The process of selecting substitutes entails engaging in discussions with field practitioners. After identifying multiple potential substitutes, the cost of the chosen substitute is evaluated.

## Method validation

### Monitoring of cost analysis

Regarding building construction, this structure's foundation is built using drilled piles or pillars, while the upper part is composed of solid concrete. The bathrooms in the classroom are equipped with uniform tiles that vary in size based on the available space on each floor. To cover ceilings, utilize exposed concrete and gypsum board. To safeguard walls, apply paint to the exterior of light brick walls. Opt for a color-protected light-colored brick material for the interior walls. Engineered doors are utilized for the doors, while aluminum frames with laminated glass are utilized for the windows. The contractor's scope of work includes mechanical, electrical, architectural, and structural tasks. The overall expenditure for constructing this project will amount to IDR 54,486.55 million. The cost, weight, and budget plan are presented in Table 1.

**Table 1:** Summary of cost weight and budget plan.

Works description	Weight (%)	Cost (millions IDR)
Structural works	42 %	23,093.24
Architectural works	35 %	18,891.52
Mechanical and electrical works	23 %	12,501.79

Total cost	100 %	54,486.55

### Eligibility test for value engineering implementation

Value engineering is employed in construction projects where there is potential for cost reduction upon completion and a substantial financial impact. After calculating and comparing the cost weights of the tasks, it was found that the structural work has the highest cost weight, which is 39.0 %. The cost weights assigned to each activity are presented in Table 1, providing a comprehensive breakdown.

After summarizing the job cost weight, the next step is to conduct an eligibility test for value engineering applications. This test involves analyzing Pareto diagrams and using a cost resolution model. Among all the tasks, structural work is the costliest. To proceed, it is necessary to enumerate the structural tasks based on their respective building expenses, commencing with the most costly work as indicated in Table 2 and subsequently descending to the least expensive tasks.

**Table 2:** Structural work summary.

Works Item	Cost (million IDR)	Structural Cost Component
Slab	5581.21	33 %
Beam	3602.15	22 %
Column	3231.40	19 %
Foundation	1901.63	11 %
Hallway	1172.36	7 %
Stair	892.18	5 %
Lift & Dog House	335.89	2 %

Total	16,716.81	100 %

The Pareto diagram is derived from analysis conducted on beams, floor slabs, and columns, which are the costliest components of the project. Hence, it is necessary to replace the material to decrease expenses. Select one of the three works to analyze the replacement of the formwork material.

According to the previous summary, the three most significant tasks will be undertaken because they have the potential to generate savings, as demonstrated by Fig. 4 for the Pareto model in relation to structural work. Table 3 displays the cost of conventional column formwork, while Table 4 presents the cost of semi-system column formwork.

**Table 3:** Conventional column formwork results.

Materials	Coefficient	Unit	Unit Price (thousand IDR)	Total cost (thousand IDR)
Pinewood	0.045	m^3^	3900	175.5
Nail	0.400	kg	22	8.8
Formwork Oil	0.200	L	21	4.20
Plywood 9 Mm	0.035	sheet	221	7.74
Volume of Ingredients				196.23
For 2 X Used				98.12
Wages:				
Workers	0.300	man.day	125	37.5
Carpenters	0.150	man.day	135	20.25
Foreman	0.015	man.day	140	2.10
others	0.015	man.day	145	2.18
Number of Workers				62.03
Tools:				
Scaffolds	1.0	package	20	20.0
Quantity of equipment				20.0

Total				180.14

**Table 4:** Semi-system column formwork results.

Material	Coefficient	Unit	Unit Price (thousand IDR)	Total cost (thousand IDR)
Woodcase	0.040	m^3^	2200	88.00
Phenol/Tegofilm	0.048	sheet	450	21.60
Formwork Oil	0.200	L	21	4.20
Nail	0.046	kg	22	1.01
Dynabolt	8.000	piece	5	40.00
Volume of Ingredients				154.81
For 5 X Used				30.96
Wages:				
Workers	0.300	man.day	125	37.5
Carpenters	0.150	man.day	135	20.25
Foreman	0.015	man.day	140	2.10
Others	0.015	man.day	145	2.18
Number of Workers				62.03
Tools:				
Scaffolds	1.0	package	20	20.00
Quantity of Equipment				20.00

Total				112.98

The unit pricing for the Psychology building construction project is derived from the initial cost analysis. The cost of the conventional column formwork is IDR 180,140 per square meter. Table 5 displays the cost of conventional column formwork, while Table 6 presents the cost of semi-system column formwork.

**Table 5:** Conventional beam formwork results.

Materials	Coefficient	Unit	Unit Price (thousand IDR)	Total cost (thousand IDR)
Pinewood	0.048	m^3^	3900	187.20
Nail	0.400	kg	22	8.80
Formwork Oil	0.200	L	21	4.20
Plywood 9 Mm	0.350	sheet	221	77.35
Volume of Ingredients	0.998			277.55
For 2 X Used				138.78
Wages:				
Workers	0.300	man.day	125	37.50
Carpenters	0.150	man.day	135	20.25
Foreman	0.015	man.day	140	2.10
Others	0.015	man.day	145	2.18
Number of Workers	0.300			62.03
Tools:				
Scaffolds	1.0	package	20	20.0
Quantity of Equipment				20.0

Total				220.80

**Table 6:** Semi-system beam formwork results.

Materials	Coefficient	Unit	Unit Price (thousand IDR)	Total cost (thousand IDR)
Woodcase	0.05	m^3^	2200	110
Phenol/Tegofilm	0.043	sheet	450	19.35
Formwork Oil	0.2	L	21	4.2
Nail	0.046	kg	22	1.012
Dynabolt	8	piece	5	40.0
Volume of Ingredients				174.56
For 5 X Used				34.91
Wages:				
The Workers	0.3	man.day	125	37.5
The Carpenters	0.15	man.day	135	20.25
The Foreman	0.015	man.day	140	2.10
The Men	0.015	man.day	145	2.18
Number of Workers	0.3			62.03
Tools:				
Scaffolds	1	package	20	20
Quantity of Equipment				20

Total				116.94

By replacing the conventional materials of plywood and nails with phenol film/tego film and dynabolt, the modified formwork system results in a cost of IDR 112,980/m^2^. There are five potential applications for this formwork. Table 5 displays the cost of conventional beam formwork, while Table 6 presents the cost of semi-system beam formwork.

The unit pricing is derived from the initial cost analysis of the Psychology Building construction project. The cost for the conventional beam formwork is IDR 220,800 per square meter, and it can be reused twice.

**Fig. 1:**
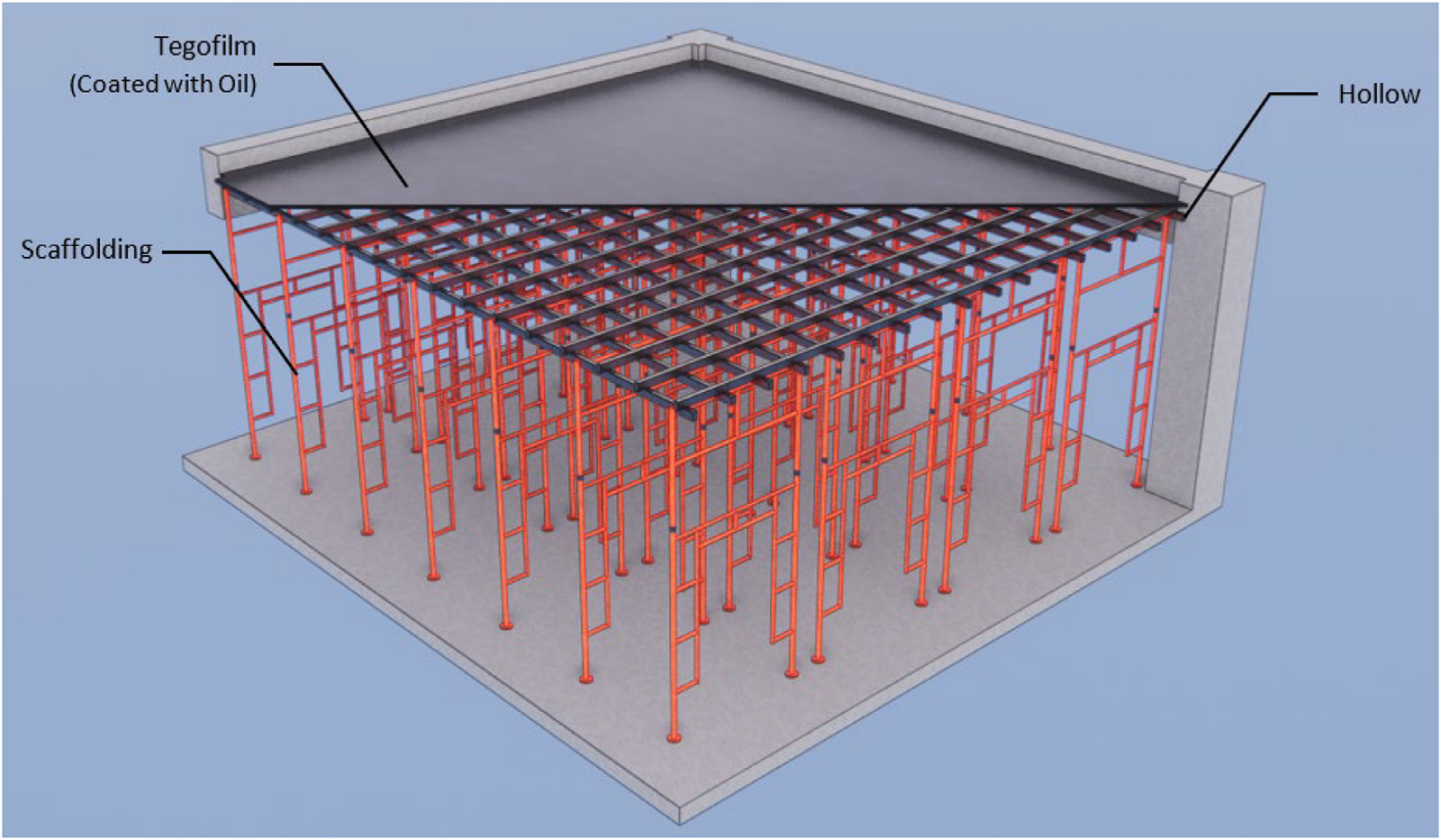
Slab semi-system formwork.

The only modification made to this semi-system formwork in Fig. 1 is the substitution of materials used in the traditional formwork. The initial use of plywood and nails resulted in an analytical cost of IDR 116,940 per square meter. Currently, phenolic film/tego film and Dyna Bolt are employed instead. This formwork can be utilized for a maximum of five instances. Table 7 displays the cost of conventional slab formwork, while Table 8 presents the cost of semi-system slab formwork.

**Table 7:** Conventional slab formwork results.

Materials	Coefficient	Unit	Unit Price (thousand IDR)	Total cost (thousand IDR)
Pinewood	0.045	m^3^	3900	175.5
Nail	0.400	kg	22	8.80
Formwork Oil	0.200	L	21	4.20
Plywood 9 Mm	0.350	sheet	221	77.35
Volume of Ingredients				265.85
For 2 X Used				132.93
Wages:				
Workers	0.300	man.day	125	37.50
Carpenters	0.150	man.day	135	20.25
Foreman	0.015	man.day	140	2.10
Others	0.015	man.day	145	2,18
Number of Workers	0.300			62.03
Tools:				
Scaffolds	1.000	package	40	40.0
Quantity of Equipment				40.0

Total				234.95

**Table 8:** Semi-system beam formwork results.

Materials	Coefficient	Unit	Unit Price (thousand IDR)	Total cost (thousand IDR)
hollow 50.50	9.394	m^3^	84	790.0
woodcase	0.005	sheet	2200	11
phenol/tego fim	0.080	L	156.25	12.5
Formwork oil	0.200	kg	221	44.20
dynabolt	3.882	piece	–	–
Volume of Ingredients				856.80
For 5 X Used				171.36
Wages:				
Workers	0.150	man.day	125	18.75
Carpenters	0.015	man.day	135	2.03
Foreman	0.015	man.day	140	2.10
Others	0.300	man.day	145	43.50
Number of workers	0.150			66.38
Tools:				
Scaffolds	1.000	package	40	40.0
Quantity of Equipment				40,0

Total				277.73

**Fig. 2:**
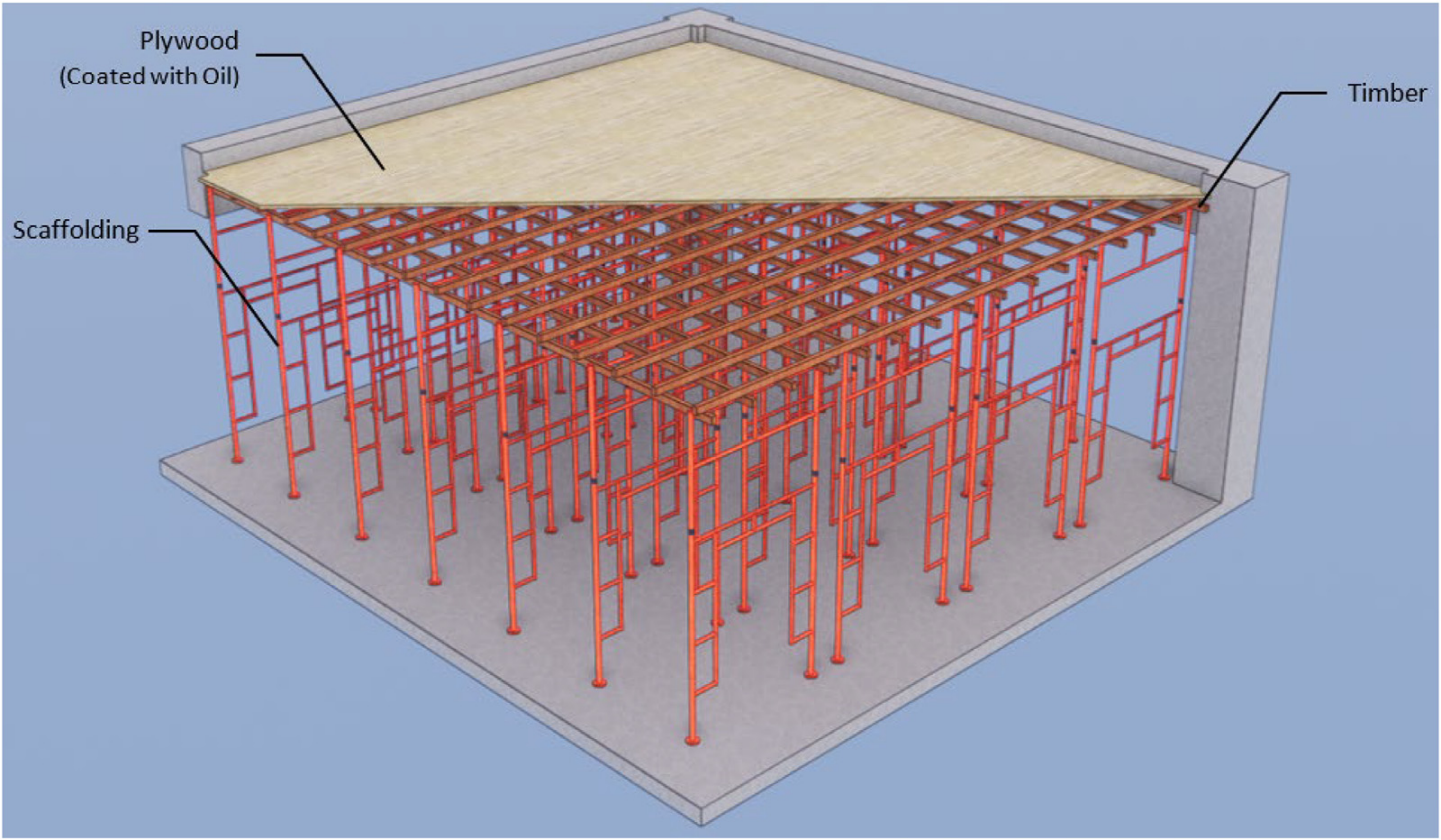
Slab conventional formwork.

The initial cost analysis conducted during the construction process formed the basic for determining the pricing of this unit. The conventional slab formwork in Fig. 2 has a cost of IDR 234,950 per square meter and can be utilized for two applications.

**Fig. 3:**
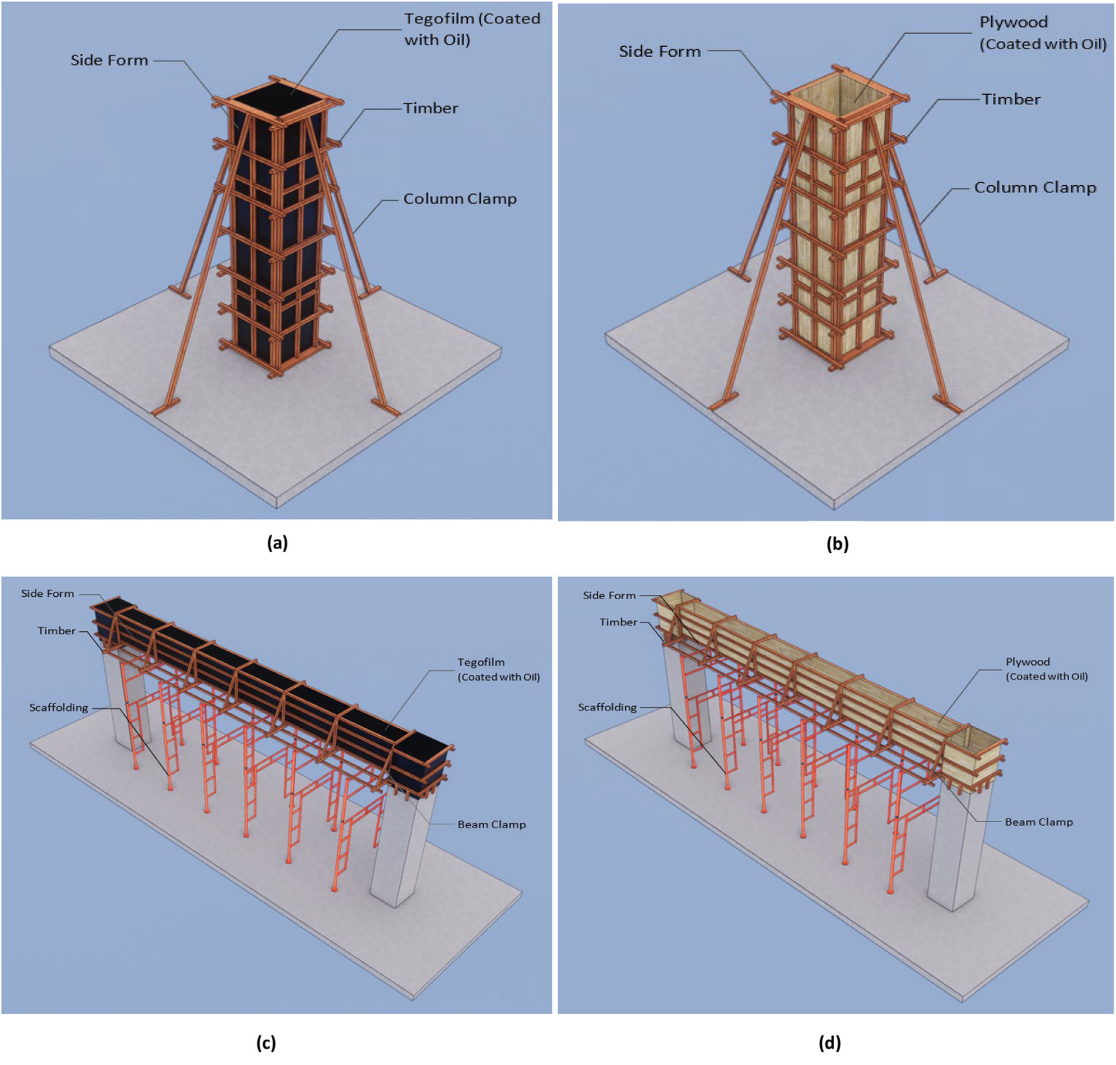
Different design formwork (a) column semi-system (b) column conventional (c) beam semi-system (d) beam conventional.

This semi-system formwork simply substitutes the materials utilized in the standard formwork. The substitution of plywood and nails with phenolic film/tego film and dynabolt resulted in an extra expense of IDR 277,730 per square meter. This formwork can be reused up to a maximum of five times (Fig. 1, Fig. 2, Fig. 3).

**Fig. 4:**
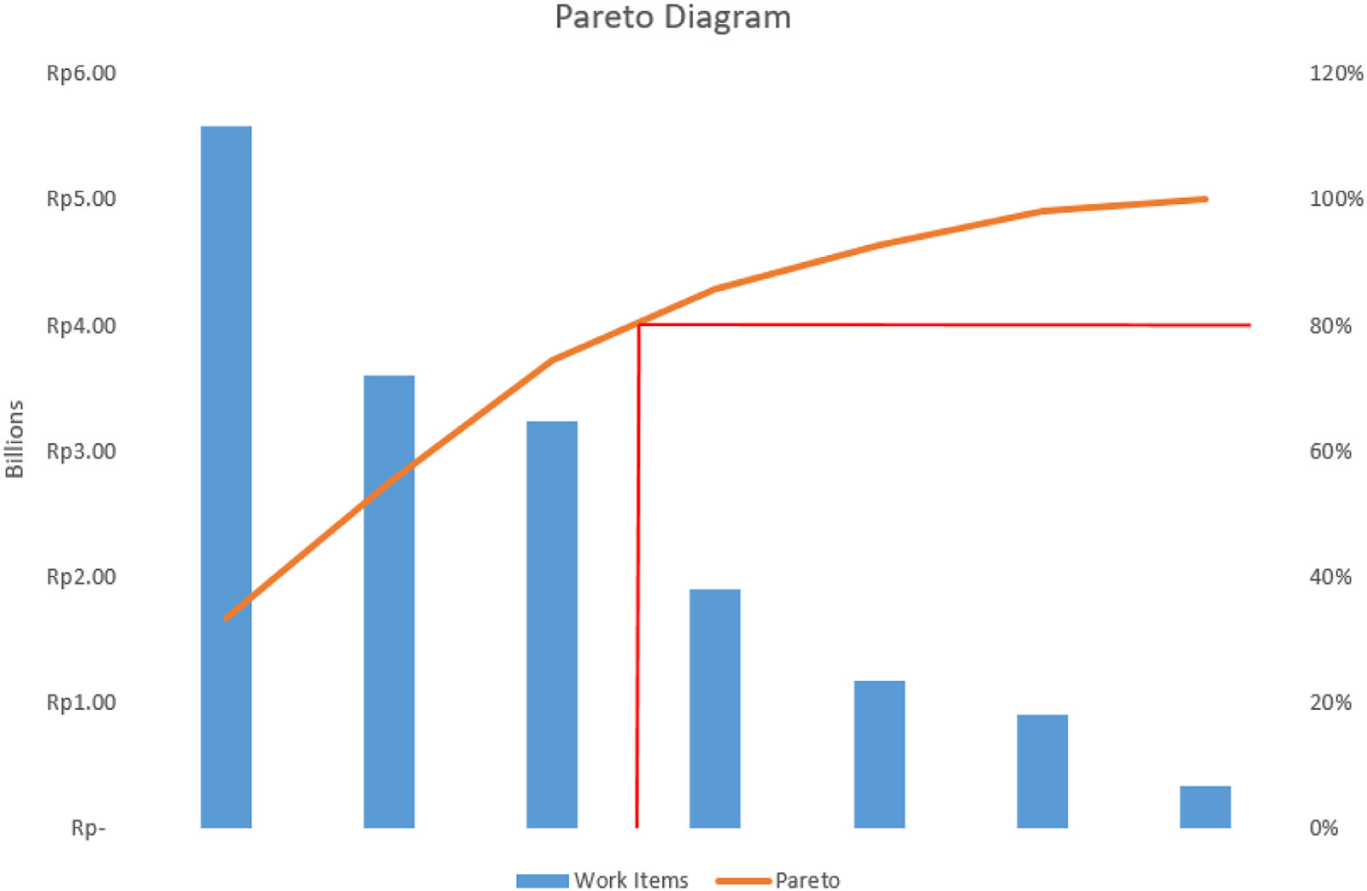
Pareto diagram of structural work.

## Summary

The conducted research can be analyzed, leading to the following conclusions. The primary expenditure in the Psychology Building is the structural work of the construction project, which includes the columns, beams, and floor slabs, as they are the essential elements. By replacing the formwork used for the columns and beams, it is possible to reduce the cost of these components. In contrast, the cost of semi-system formwork is higher than that of standard formwork specifically for floor slabs. If the formwork were substituted, it would be possible to replace the following building materials with more cost-effective alternatives:

Before implementing value engineering, the cost of column formwork was IDR 180,140/m^2^. However, if semi-system formwork had been used, the cost would have been IDR 112,980/m^2^. The semi-system formwork results in a cost savings of IDR 67,160 per square meter, making it 37 % cheaper than traditional formwork.

Prior to implementing Value Engineering, the cost of beam formwork was IDR 220,800 per square meter. However, if semi-system formwork had been utilized, the cost would have been reduced to IDR 116,940 per square meter. Consequently, the semi-system formwork achieves a cost reduction of IDR 103,860/m^2^, resulting in a 47 % decrease in expenses compared to traditional formwork. The cost of floor slab formwork was IDR 239,950/m2 before value engineering. If semi-system formwork had been used, the cost for the same area would have been IDR 269,260/m^2^. Consequently, the cost of traditional formwork is 15 % lower than that of semi-system formwork, resulting in a cost differential of 34,310/m^2^.

Hence, semi-system formwork is the optimal choice for constructing the columns and beams of this building, while traditional formwork remains the preferred option for the floor slab.

## Limitations

The limitations that this method described have certain conditions in which the method may not work, such as in infrastructure, foundations, tunnels, pavement or other flat structures. The methods only works in upper structures of framed construction of mid to high rise building, hence not include the bottom structures. The methods will not works in shear wall structures, steel composite structures, braced structures and outriggered structures.

## Ethics statements

This work do not involved human, animal nor the data collected from social media, those here we declare a statement that project data and the location has been fully anonymized.

## Supplementary material and/or additional information [OPTIONAL]

N/A

## CRediT authorship contribution statement

**Muhammad D.V. Lawdy:** Investigation, Writing – original draft, Resources, Project administration, Visualization. **Eva Arifi:** Conceptualization, Methodology, Writing – review & editing, Supervision, Resources, Validation. **Ming Narto Wijaya:** Conceptualization, Methodology, Writing – review & editing, Supervision, Resources, Validation. **Bagus Krisnawan:** Resources, Project administration, Writing – original draft, Visualization. **Taufiq Rochman:** Resources, Writing – review & editing, Supervision.

## Declaration of Competing Interest

The authors declare that they have no known competing financial interests or personal relationships that could have appeared to influence the work reported in this paper.

## Data Availability

Data will be made available on request.
